# Resveratrol Metabolism in a Non-Human Primate, the Grey Mouse Lemur (*Microcebus murinus*), Using Ultra-High-Performance Liquid Chromatography–Quadrupole Time of Flight

**DOI:** 10.1371/journal.pone.0091932

**Published:** 2014-03-24

**Authors:** Marie-Claude Menet, Julia Marchal, Alexandre Dal-Pan, Méryam Taghi, Valérie Nivet-Antoine, Delphine Dargère, Olivier Laprévote, Jean-Louis Beaudeux, Fabienne Aujard, Jacques Epelbaum, Charles-Henry Cottart

**Affiliations:** 1 EA 4463, Faculté des Sciences Pharmaceutiques et Biologiques, Université Paris Descartes, Sorbonne Paris Cité, Paris, France; 2 Mécanismes Adaptatifs et Evolution, UMR 7179, Centre National de la Recherche Scientifique, Muséum National d'Histoire Naturelle, Brunoy, France; 3 EA 4466, Faculté des Sciences Pharmaceutiques et Biologiques, Université Paris Descartes, Sorbonne Paris Cité, Paris, France; 4 Service de Biochimie, AP-HP, Hôpital Européen Georges Pompidou, Paris, France; 5 Service de Toxicologie biologique, AP-HP, G.H. Lariboisière – Saint Louis – Fernand Widal, Paris, France; 6 Service de Biochimie A, G.H. Necker - Enfants malades, AP-HP, Paris, France; 7 Centre de Psychiatrie et Neuroscience, UMR 894 Inserm, Faculté de Médecine, Université Paris Descartes, Paris, France; Charité, Campus Benjamin Franklin, Germany

## Abstract

The grey mouse lemur (*Microcebus murinus*) is a non-human primate used to study the ageing process. Resveratrol is a polyphenol that may increase lifespan by delaying age-associated pathologies. However, no information about resveratrol absorption and metabolism is available for this primate. Resveratrol and its metabolites were qualitatively and quantitatively analyzed in male mouse-lemur plasma (after 200 mg.kg^−1^ of oral resveratrol) by ultra-high performance liquid chromatography (UHPLC), coupled to a quadrupole-time-of-flight (Q-TOF) mass spectrometer used in full-scan mode. Data analyses showed, in MS^E^ mode, an ion common to resveratrol and all its metabolites: *m/z* 227.072, and an ion common to dihydro-resveratrol metabolites: *m/z* 229.08. A semi-targeted study enabled us to identify six hydrophilic resveratrol metabolites (one diglucurono-conjugated, two monoglucurono-conjugated, one monosulfo-conjugated and two both sulfo- and glucurono-conjugated derivatives) and three hydrophilic metabolites of dihydro-resveratrol (one monoglucurono-conjugated, one monosulfo-conjugated, and one both sulfo- and glucurono-conjugated derivatives). The presence of such metabolites has been already detected in the mouse, rat, pig, and humans. Free resveratrol was measurable for several hours in mouse-lemur plasma, and its two main metabolites were *trans*-resveratrol-3-O-glucuronide and *trans*-resveratrol-3-sulfate. Free dihydro-resveratrol was not measurable whatever the time of plasma collection, while its hydrophilic metabolites were present at 24 h after intake. These data will help us interpret the effect of resveratrol in mouse lemurs and provide further information on the inter-species characteristics of resveratrol metabolism.

## Introduction

Resveratrol is a dietary polyphenol compound present in numerous plants, especially grapes. *In vitro* and *in vivo* studies have highlighted that resveratrol could display properties that could be applied to human health. Among them, resveratrol acts on glucose and lipid metabolism [1] and may limit the development of cardiovascular [2], [3] and neurodegenerative [4], [5] diseases, as well as cancer [6]. Moreover, even though it is still controversial, resveratrol appears to mimic calorie restriction, at least in part, by acting through the sirtuin pathway [7]. Indeed, resveratrol can increase the lifespan of numerous species, from yeast to mice [8].

When ingested *per os*, resveratrol, which is a lipophilic compound, is well absorbed, and rapidly metabolized in the intestine and liver into hydrophilic metabolites, which are then eliminated in the urine. After oral intake, the small quantity of non-metabolized resveratrol in plasma has raised some doubts about its capacity to display physiological effects. Moreover, intestinal gut microflora may hydrogenate resveratrol's double bond to form dihydro-resveratrol (DHR), which can then be absorbed and metabolized [9]. However, the sulfo- and glucurono-metabolites of resveratrol are mainly represented in plasma and could sustain their own activities, although resveratrol metabolism may vary from one species to another [10]. Up until now, the identification and assessment of resveratrol metabolites has been performed in several species, such as the mouse [11], rat [12], dog [13], pig [14], and humans [15], [16].

The grey mouse lemur (*Microcebus murinus*), a small heterothermic primate, is a relevant model for studying ageing [17], [18], [19], as it has a median lifespan of 5.7 years for males, and a maximal lifespan of 11 years. These data are based on those from the Brunoy breeding colony [18], in which mouse-lemurs lived for two-to-three times longer than mammals of equivalent body mass.

Grey mouse lemurs are phylogenetically closer to humans than rodents; therefore, they are used to test new dietary or pharmacological strategies to improve lifespan and decrease age-associated pathologies [20], [21]. Available data show that resveratrol treatment can affect metabolism [20], [22], cognitive performance [17], and the sleep–wake cycles [23] of this lemur. More precisely, this primate has marked seasonal metabolic rhythms: it has a resting metabolic rate and body weight that fluctuates seasonally. In 2010, a study by Dal-Pan et al. reported on the effect of acute resveratrol treatment (200 mg.kg^−1^.day^−1^) [22]. The authors regularly measured body temperature, bodyweight gain, and resting metabolic rate of treated animals. After a 4-week of treatment, they found an immediate effect: the treated animals had reduced food intake by 13% and had increased their resting metabolic rate (which was part of their energy expenditure) by 29%. Thus, ingestion of resveratrol allowed these animals to significantly reduce weight, while they would normally tend to gain weight and store-up energy before the breeding season. These results provide new information on the effects of resveratrol on energy metabolism and the control of body weight in primates. However, no information on resveratrol absorption and metabolism in *Microcebus murinus* is currently available.

The aim of the present study was to identify and assess resveratrol metabolites in the plasma of mouse lemurs after oral intake of 200 mg.kg^−1^ of resveratrol, using ultra-high performance liquid chromatography (UHPLC) coupled to a high-resolution mass-spectrometer, quadrupole-time of flight (Q-TOF) [11].

## Materials and Methods

### 1. Chemicals and materials

**Figure 1:**
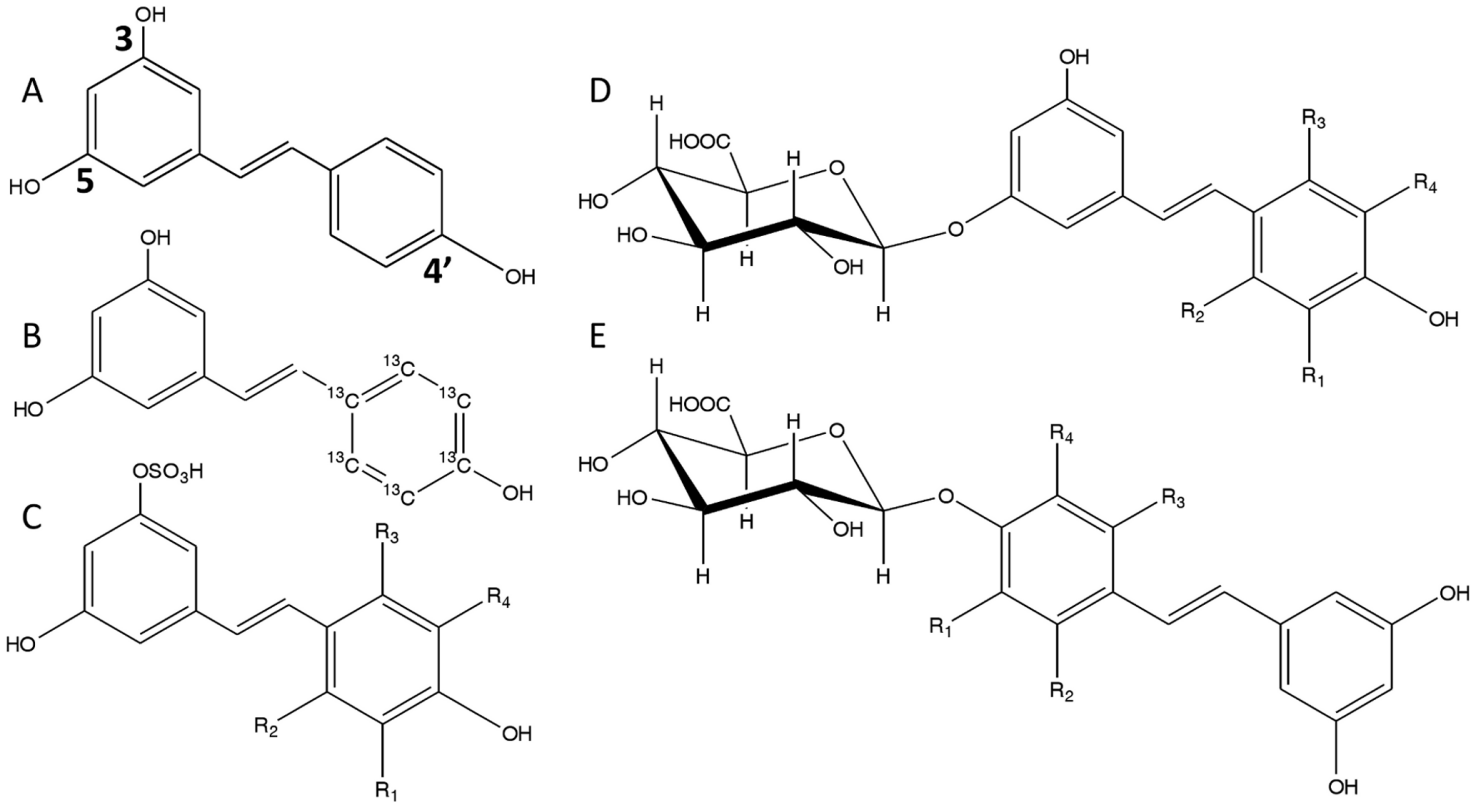
Chemical structure of A: *Trans*-resveratrol; B: *trans*-resveratrol-^13^C_6_; C: *trans*-resveratrol-3-sulfate (R1 = R2 = R3 = R4 = H) or *trans*-resveratrol-3-sulfate-D4 (R1 = R2 = R3 = R4 = D); D: *trans*-resveratrol-3-O-ß-D-glucuronide (R1 = R2 = R3 = R4 = H) or *trans*-resveratrol-3-O-ß-D-glucuronide-D_4_ (R1 = R2 = R3 = R4 = D); E: *trans*-resveratrol-4′-O-ß-D-glucuronide (R1 = R2 = R3 = R4 = H) or *trans*-resveratrol-4′-O-ß-D-glucuronide-D_4_ (R1 = R2 = R3 = R4 = D).

A reference standard (Fig. 1) of *trans*-resveratrol (99%, w/w) was purchased from Sigma (Saint-Quentin-Fallavier, France). *Trans*-resveratrol-^13^C6 was obtained from LGC Standard (Molsheim, France). Reference standards (Fig. 1) of *trans*-resveratrol-3-O-ß-D-glucuronide, *trans*-resveratrol-4′-O-ß-D-glucuronide, *trans*-resveratrol-3-sulfate, *trans*-resveratrol-3-O-ß-D-glucuronide-D4, *trans*-resveratrol-4′-O-ß-D-glucuronide-D4, and *trans*-resveratrol-3-sulfate-D4 were provided by Spi Bio, Bertin Pharma (Montigny le Bretonneux, France). Because of the low stability of resveratrol and its derivatives in ultraviolet light [24], all reference analytes and internal standards were stored in the dark at −20°C.

HPLC-grade acetonitrile (ACN) and methanol (MeOH) were purchased from Merck (Nogent-sur-Marne, France). Formic acid (≥99% w/w), HPLC grade, was obtained from Fischer Scientific (Illkirch, France). All other chemicals were of analytical grade, and the solvents used were of chromatographic grade. All water was prepared with a Milli-Q water purification system (Millipore, Molsheim, France). Resveratrol (98.3%, w/w, Sequoia Research Products, UK) was used for the gavage of mouse lemurs.

### 2. Study design

The grey mouse lemurs were born in the laboratory breeding colony of Brunoy (Muséum National d'Histoire Naturelle, UMR 7179 CNRS/MNHN, France; Agreement DDPP # D91-114-1). All experiments were performed in accordance with the Principles of Laboratory Animal Care (National Institutes of Health publication 86-23, revised 1985) and the European Communities Council Directive (86/609/EEC). Regarding the environmental enrichment and housing of the grey-mouse lemurs used in this study, in order to minimize social influences during the different experiments, animals were housed individually in 1 m^3^ cages, provided with two wooden nests and separated from each other by metallic partitions. The grey mouse lemur is a nocturnal primate that shows high levels of locomotor activity, thus we provided in each cage, many natural tree branches. The research was conducted under the authorization number 91–305 from the “Direction Départementale des Services Vétérinaires de l'Essonne” and the Internal Review Board of the UMR 7179. In accordance with the recommendations of the Weatherall report, “The use of non-human primates in research”, special attention was paid to the welfare of the animals during this work to minimize nociception (Weatherall FRS D. 2006. The use of non-human primates in research, The Weatherall report).

General conditions of captivity were consistently maintained. Animals were exposed to ambient room temperature (24–26°C) and a relative humidity of 55%. Animals were fed *ad libitum* every 2 days: fresh fruits (apples and banana), and a homemade mixture of white cheese, ginger bread, baby cereal, milk, and egg were provided. Water was given *ad libitum*. These mouse lemurs (*n* = 20), weighing 98.2±42.3 g, were divided into five groups. One group was not treated with resveratrol and was used as a control (*n* = 3); the other four groups were given resveratrol and plasma was collected at 1 (*n* = 4), 2 (*n* = 5), 6 (*n* = 4), or 24 h (*n* = 4) after intake of resveratrol. All lemurs used in this study were males. It should be noted that NO grey mouse lemur has been sacrificed in this study.

There is a lack of published data on the metabolism of phenolic substances, and particularly polyphenols, in mouse lemurs, although experiments are ongoing in the wild in Madagascar to assess the amounts of different phenolic substances ingested daily and seasonally in these primates. Up until now, we have had no evidence indicating any difference in the metabolism of males and females.

The mouse lemurs in our study were given 200 mg.kg^−1^ of resveratrol by gavage. A dose of 200 mg.kg^−1^ of body weight has been previously given to grey mouse lemurs in the Restrikal project; the first results exhibited a physiological effect from this dose of resveratrol [20], [21]. In the present study, resveratrol (20 mg.mL^−1^) was administered within the homemade food mixture. Blood samples were taken via the saphenous vein, without anesthesia, at the end of the resting phase but before food became available. Blood samples (100–150 µL) were collected in tubes containing EDTA. They were then centrifuged at 2000 *g* at 4°C for 30 min. Before storage of the plasma at −80°C, the samples underwent a second centrifugation at 2000 *g* at 4°C for 10 min.

### 3. Plasma samples used for quantification

The blank plasma samples used for calibration standards and quality control samples were obtained from Swiss mice (*n* = 20), provided by Janvier (Le Genest-St-Isles, France), and used for a study entitled “Measurement of resveratrol in mouse tissues by UHPLC-Q-TOF,” which has been approved by the Ethical Committee of the University Paris Descartes, and is registered as number CEEA34.CHC.043.11.

### 4. Quantification and study design

The method used to quantify resveratrol and its sulfo- and glucurono-derivatives has been previously validated in mouse plasma and has been described in detail elsewhere [11]. Briefly, initial standard-stock solutions of *trans*-resveratrol, *trans*-resveratrol-4′-O-ß-D-glucuronide, *trans*-resveratrol-3-O-ß-D-glucuronide, and *trans*-resveratrol-3-sulfate, as well as initial corresponding internal standard-stock solutions, were prepared in MeOH at 5 mg.mL^−1^. For calibration standards, different stock solutions that combined all four compounds were then prepared in a mixture of ACN/water (20/80; V/V) at concentrations of 20 times higher than those of the calibration standards, i.e., between 0.5 and 320 µmol.L^−1^. The final internal standard-stock solution, combining all four internal standards, with concentrations of 40 µmol.L^−1^ each of *trans*-resveratrol-3-sulfate-D_4_ and *trans*-resveratrol-^13^C_6_, and 200 µmol.L^−1^ each of *trans*-resveratrol-4′-O-ß-glucuronide-D_4_ and *trans*-resveratrol-3-O-ß-glucuronide-D_4_, was prepared in the ACN/water (20/80; V/V) mixture.

### 5. Extraction of resveratrol and its metabolites from plasma

Plasma samples were prepared according to Menet *et al.* [11]. Briefly, 50 µL of untreated mouse plasma spiking by analyte standards or 50 µL of treated lemur plasma, were vortex-mixed with the internal standard solution and 200 µL of ACN for 20 s. They were then let to stand for 20 min and centrifuged at 1000 *g* for 10 min. Supernatants were separated and evaporated to dryness using a Speed Vac Concentrator (Savant, SPD131DDA, Thermo Fischer Scientific, Les Ulis, France). The dried extracts were reconstituted in 50 µL of ACN/water/formic acid (20/80/0.1; V/V/V).

### 6. Ultra-high performance liquid chromatography–mass spectrometry conditions and instrumentation

The conditions for analyses were the same as described by Menet et al. [11]. Samples were injected into an Acquity UPLC system (Waters, Manchester, UK), equipped with an Acquity UHPLC BEH C18 column (50×2.1 mm inner diameter, particle size 1.7 µm), which was purchased from Waters (Guyancourt, France). The mobile phase consisted of mixture of water and ACN. It was operated with a flow-rate of 0.4 mL/min in gradient mode, at a temperature of 30°C. Mass spectra were recorded with a Waters Synapt G2 HDMS mass spectrometer (Waters, Manchester, UK). Measurements were performed using negative electrospray ionization (ESI) in full-scan data-acquisition mode. The parameters of the electrospray interface were optimized to provide the highest abundance of the [M–H]^−^ ion for all analytes and internal standards.

MS^E^ method (centroide) was used to identify the metabolites. The scan time was set at 0.1 s. All data were lock-mass connected with the pentapeptide–leucine enkephalin, Tyr–Gly–Gly–Phe–Leu ([M–H]^−^ at *m/z* 554.2615), in a ACN/water/formic acid (50/50/0.1; V/V/V) solution (2 ng.µL^−1^). Calibration of the Synapt G2 mass spectrometer in the range of *m/z* 50–1200 was performed using a solution of sodium formate (5 mmol.L^−1^) in 2-propanol/water (9/1; V/V).

For each compound, the [M–H]^−^ ion was used for quantification, according to the quantification method described by Menet et al. [11]. Indeed, this study has shown that, such as Stark et al. [25], the performances of the UHPLC–Q-TOF (i.e. high-resolution mass spectrometer) combined with SIDA (stable isotope dilution analysis), compared with HPLC-MRM-MS combined with SIDA, are similar in terms of quantification.

Data processing was performed using MassLynx software (V 4.1, SCN 779, Waters, Manchester, UK) and the elemental composition tool. The QuantLynx (V 4.1, SCN 803, Waters) was used to detect and integrate the peaks of the [M-H]^−^ ions at *m/z* 227.07 (*trans*-resveratrol), *m/z* 233.09 (*trans*-resveratrol-^13^C_6_), *m/z* 403.10 (*trans*-resveratrol-3-O-ß-D-glucuronide, and *trans*-resveratrol-4′-O-ß-D-glucuronide); *m/z* 407.13 (*trans*-resveratrol-3-O-ß-D-glucuronide-D_4_, and *trans*-resveratrol-4′-O-ß-D-glucuronide-D_4_); *m/z* 307.03 (*trans*-resveratrol-3-sulfate), *m/z* 311.05 (*trans*-resveratrol-3-sulfate-D_4_), *m/z* 483.06 (*trans*-resveratrol-glucuronide-sulfate derivatives), *m/z* 485.08 (DHR-glucuronide-sulfate derivative), *m/z* 405.12 (DHR-glucuronide), and *m/z* 309.04 (DHR-sulfate).

### 7. Determination of resveratrol and its metabolites in plasma samples from resveratrol-treated grey mouse lemurs

Plasma samples from mouse grey lemurs treated with *trans*-resveratrol were assayed and the concentrations of *trans*-resveratrol and its metabolites measured from the peak-area ratios of the analytes and internal standards. These ratios were used to calculate the concentrations of compounds within the plasma specimens from the calibration curves, which were prepared as described above. Quality control samples were included in each analytical batch to check for calibration, accuracy, and precision.

The retention times of *trans*-resveratrol-glucuronide-sulfate derivatives (2.05 and 2.32 min) as well as the DHR-glucuronide-sulfate derivative (2.05 min) were close to that of *trans*-resveratrol-4′-O-ß-D-glucuronide (2.28 min). As the standards and internal standards of these compounds are not available, their concentrations were estimated by using *trans*-resveratrol-4′-O-ß-D-glucuronide-D_4_ as internal standard and the corresponding calibration curve. Concentrations were expressed as *trans*-resveratrol 4′-O-ß-D-glucuronide equivalents.

The retention time of DHR-glucuronide (3.49 min) was close to that of *trans*-resveratrol-3-O-ß-D-glucuronide-D_4_ (2.93 min), and the retention time for DHR-sulfate (3.88 min) was close to that of *trans*-resveratrol-3-sulfate (3.86 min). As the standards and internal standards for DHR derivatives are not available, their concentrations were estimated using *trans*-resveratrol-3-O-ß-D-glucuronide-D_4_ and *trans*-resveratrol-3-sulfate-D_4_ as internal standards for DHR-glucuronide and DHR-sulfate respectively and the corresponding calibration curves. Concentrations were, respectively, expressed as equivalents of *trans*-resveratrol-3-O-ß-D-glucuronide and *trans*-resveratrol-3-sulfate.

## Results and Discussion

### 1. Identification of resveratrol and its metabolites

Resveratrol and its metabolites have been identified through UHPLC coupled to Q-TOF mass spectrometry (Synapt^R^) in MS^E^ mode. The latter allows accurate monoisotopic masses (lower than 10 ppm) to be recorded at high- and low-collision energies successively. The first recording allows the *m/z* ratio of the pseudo-molecular ion [M–H]^−^ to be obtained, while the second recording displays the *m/z* ratio of the daughter ions. Thus, UHPLC–Q-TOF–MS^E^ is a powerful tool to analyze complex mixtures, especially biological fluids, and enables to obtain the elucidation of elemental composition and fragmentation information [26]. Herein, the composition of mouse-lemur plasma was analyzed with this new approach, which permitted the structures of several endogenous components to be confirmed in one analytical run by the simultaneous acquisition of exact masses of precursor and daughter ions.

**Figure 2:**
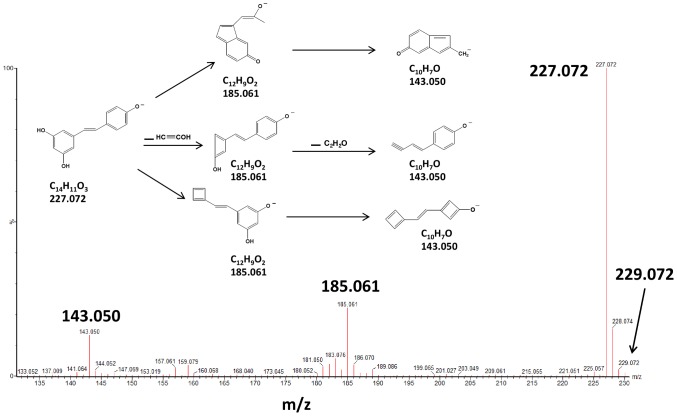
High-collision energy spectrum of ions specific to resveratrol, *m/z* 227.072, showing the daughter ions formed.

The identification of resveratrol and resveratrol metabolites in samples was made in two steps. First at all, UPLC–Q-TOF–MS^E^ analysis of standards, *trans*-resveratrol-3-O-ß-D-glucuronide, *trans*-resveratrol-4′-O-ß-D-glucuronide, *trans*-resveratrol-3-sulfate, and *trans*-resveratrol enabled to obtain their retention time and their spectra at low- and high-collision energies. *Trans*-resveratrol-4′-O-ß-D-glucuronide had a retention time of 2.28 min, *trans*-resveratrol-3-O-ß-D-glucuronide of 2.93 min, *trans*-resveratrol-3-sulfate of 3.86 min, and *trans*-resveratrol of 4.35 min. Figure 2 shows the high-collision energy spectrum obtained after the fragmentation of the pseudo-molecular ion *m/z* 227.072 (as a result of resveratrol ionization in the ESI source) and the *m/z* ratios of the daughter ions, 143.050 and 185.061, which correspond to the elemental composition of each ion, C_10_H_7_O (143.050) and C_12_H_9_O_2_ (185.061), respectively. The same fragmentation pattern was encountered for hydrophilic metabolites.

**Figure 3:**
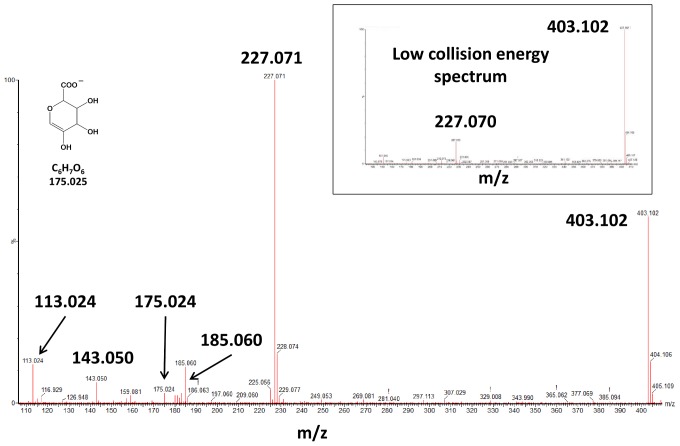
Low- and high-collision energy spectra of ions specific to resveratrol-glucuronide, *m/z* 403.102.

Figure 3 shows spectra obtained at low- and high-collision energies for the pseudo-molecular ion *m/z* 403.102, which is characteristic of monoglucurono-conjugated derivatives. The low-energy spectrum shows that the glucurono moiety was lost easily, with the emergence of a *m/z* 227.070 ion. The high-collision energy spectrum shows the presence of all daughter ions that are the characteristic of *m/z* 227.071, i.e., *m/z* 143.050 and 185.060, together with the ions characteristic of glucurono-conjugated derivatives, *m/z* 175.024 (C_6_H_7_O_6_) and 113.024 (C_5_H_5_O_3_) resulting from the rearrangement of the previous ion *m/z* 175.024 (Fig. 3) [27]. The same kind of spectra has been obtained with *trans*-resveratrol-3-sulfate, with the loss of the sulfo moiety and fragmentation of the resulting *m/z* 227.072 ion.

The results for precursor and daughter ions in the four standards studied are reported in Table 1. The averages of *m/z* values were determined after five injections of standard mixtures at 6, 8, and 16 µmol.L^−1^ in UHPLC–Q-TOF. The mass accuracy for all ions was lower than 10 ppm, from lower than 1 to 8.8 ppm. These results highlight that the accuracy of mass determination of the daughter ions enabled their structures to be confirmed, as reported in the literature [28], [29]. These data were compared to those recorded after analyzing the treated mouse lemur plasmas where *trans*-resveratrol-3-O-ß-D-glucuronide, *trans*-resveratrol-4′-O-ß-D-glucuronide, *trans*-resveratrol-3-sulfate, and resveratrol were found (Table 1). The mass accuracy for all ions was lower than 10 ppm. Thus, the metabolites were identified by their retention time and the presence of specific ions in their high-collision energy spectrum, i.e., *m/z* 143.050, 185.060, and 227.071, except for *trans*-resveratrol-4′-O-ß-D-glucuronide. For the latter, in the sample plasma chromatograms, the intensity of the pseudo-molecular ion *m/z* 403.099 was low; consequently, the daughter ions, *m/z* 143.050 and 185.060, were not detected. Therefore, this compound was identified by the presence of ions *m/z* 403.099, 227.069, and 113.023, with accuracy lower than 10 ppm, i.e., 9.9, 8.8, and 8.8 ppm, respectively. Generally, accuracy was lower (values close to 10 ppm) when the ion intensity was low.

**Table 1:** *m/z* average and accuracy (ppm) for precursor and daughter ions of resveratrol and their metabolites.

Compound	Ion Formula	Theoretical mass	Standard *m/z* (ppm)/metabolite *m/z* (*n*, ppm)
RGS	C_20_H_19_O_12_S	483.059	483.060 (34, 2)
	C_20_H_19_O_9_	403.103	403.099 (32, −9.9)
	C_14_H_11_O_6_S	307.027	307.029 (32, 6.5)
	C_14_H_11_O_3_	227.071	227.069 (30, −8.7)
	C_12_H_9_O_2_	185.060	185.061 (21, 5.4)
	C_10_H_7_O	143.050	143.049 (16, −7.0)
	C_5_H_5_O_3_	113.024	113.023 (24, −8.8)
4′RG	C_20_H_19_O_9_	403.103	403.102 (−2.5)/403.099 (20, −9.9)
	C_14_H_11_O_3_	227.071	227.070 (−4.4)/227.069 (19, −8.8)
	C_12_H_9_O_2_	185.060	185.061 (7, 5.4)
	C_10_H_7_O	143.050	143.049 (5, −6.9)
	C_5_H_5_O_3_	113.024	113.023 (−8.8)/113.023 (20, −8.8)
3RG	C_20_H_19_O_9_	403.103	403.103 (0.5)/403.102 (34, −2.5)
	C_14_H_11_O_3_	227.071	227.070 (−4.4)/227.072 (34, 4.4)
	C_12_H_9_O_2_	185.060	185.061 (5.4)/185.058 (27, −9.9)
	C_10_H_7_O	143.050	143.050 (1.1)/143.049 (27, −7.0)
	C_5_H_5_O_3_	113.024	113.025 (8.8)/113.023 (28, −8.8)
RS	C_14_H_11_O_6_S	307.027	307.028 (4.0)/307.028 (34, 3.6)
	C_14_H_11_O_3_	227.071	227.071 (1.0)/227.073 (34, 8.8)
	C_12_H_9_O_2_	185.060	185.059 (−3.6)/185.060 (27, 1.4)
	C_10_H_7_O	143.050	143.050 (0.9)/143.050 (27, −1.1)
R	C_14_H_11_O_3_	227.071	227.070 (−4.9)/227.071 (28, 0.47)
	C_12_H_9_O_2_	185.060	185.060 (1.2)/185.061 (28, 3.3)
	C_10_H_7_O	143.050	143.050 (0.6)/143.049 (28, −5.2)
DHRGS	C_20_H_21_O_12_S	485.076	485.076 (8, 0.7)
	C_20_H_21_O_9_	405.119	405.118 (8, −2.4)
	C_14_H_13_O_6_S	309.044	309.044 (8, 0.9)
	C_14_H_13_O_3_	229.087	229.085 (8, −8.7)
	C_7_H_7_O_2_	123.045	123.045 (4, −0.8)
	C_5_H_5_O_3_	113.024	113.023 (7, −8.8)
DHRG	C_20_H_21_O_9_	405.119	405.118 (8, −2.4)
	C_14_H_13_O_3_	229.087	229.085 (8, −8.7)
	C_7_H_7_O_2_	123.045	123.044 (3, −8.1)
	C_5_H_5_O_3_	113.024	113.025 (8, 8.8)
DHRS	C_14_H_13_O_6_S	309.044	309.045 (8, 3.5)
	C_14_H_13_O_3_	229.087	229.086 (8, −4.4)
	C_7_H_7_O_2_	123.045	123.045 (8, −0.8)

RGS = *trans*-resveratrol-glucuronide-sulfate; 4′RG = *trans*-resveratrol-4′-O-ß-glucuronide; 3RG = *trans*-resveratrol-3-O-ß-glucuronide; RS = *trans*-resveratrol-sulfate, R = resveratrol; DHRGS = DHR-glucuronide-sulfate; DHRG = DHR-glucuronide; DHRS = DHR-sulfate.

Standard samples: 3×5 injections; plasma samples: 17×2 injections.

*n*: Number of injections where an ion was found.

**Figure 4:**
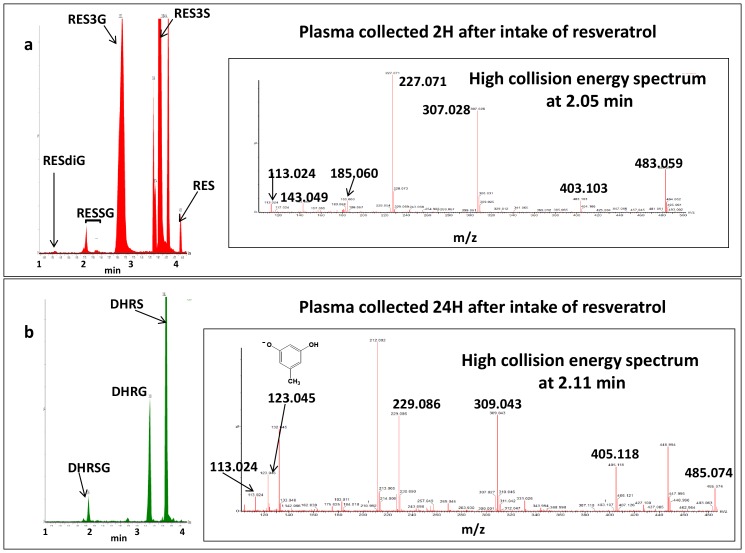
Chromatograms: variations in the intensity of *m/z* 227.07 (a) and *m/z* 229.087 (b) vs. time. **a**: Time 2 h after intake of resveratrol. High-collision energy spectrum at 2.05 min. **b**: Time 24 h after intake of resveratrol. High-collision energy spectrum at 2.11 min.

Our study has shown the easy loss of glucurono- and sulfo- moieties of conjugated derivatives, with the emergence of the *m/z* 227.072 ion in low-collision energy spectra. This result was used to find other possible glucurono- and/or sulfo-conjugated derivatives in mouse-lemur plasma, according to a semi-targeted mode. The variation in intensity of the *m/z* 227.071 ion obtained at high-collision energy, versus time is shown in figure 4a, for plasma obtained 2 h after intake. This chromatogram shows the presence of *trans*-resveratrol-3-O-ß-D-glucuronide and *trans*-resveratrol-3-sulfate, together with other glucurono- and/or sulfo-conjugated derivatives with retention times close to 1.30 and 2 min, respectively. Figure 4a also shows the high-collision energy spectrum at 2.05 min. Ions corresponding to a both glucurono- and sulfo-conjugated derivative are noted *m/z* 483.059, together with *m/z* 307.028 and 403.103, appeared after the loss of glucurono- and sulfo- moieties, respectively. The daughter ions specific to resveratrol, *m/z* 143.049 and 185.060, were also detected at the same retention time. The 2.32-min spectrum displays the same characteristics. Table 1 shows the averages *m/z* ratio and a satisfactory accuracy value (<10 ppm) for each ion. The 1.30-min spectrum shows the *m/z* 579.135 ion after ionization of a di-glucurono-conjugated derivative (spectrum not shown). The ion intensity is weak; consequently, the daughter ions, *m/z* 403.101, 227.072, and 113.024, appear in the high-energy collision spectrum, but not *m/z* 143.050 and 185.060. The identification of this compound was confirmed by the accuracy of the *m/z* values, i.e., 0.9, −4.7, and −4.4 ppm for, respectively, *m/z* 579.135, 403.103, and 227.071. This derivative was present in mouse-lemur plasma, but at a very low concentration. This was confirmed by the quantitative study described in the next paragraph; the concentrations of this metabolite in the plasmas of all mouse lemurs were below the lower limit of quantification and, therefore, are not listed in Tables 1 and 2.

**Table 2:** Concentrations of *trans*-resveratrol and its hydrophilic metabolites and hydrophilic metabolites of dihydro-resveratrol (DHR).

	1 h (*n* = 4)	2 h (*n* = 5)	6 h (*n* = 4)	24 h (*n* = 4)
*Trans*-resveratrol glucuronide sulfate 1	1.87 (57.18)	3.89 (120.88)	1.25 (27.44)	0.49 (60.55)
	[0.78–3.34]	[0.91–12.24]	[0.93–1.72]	[0.25–0.92]
*Trans*-resveratrol glucuronide sulfate 2	0.41 (39.99)	0.73 (102.10)	0.30 (7.45)	0.31 (59.34)
	[0.22–0.61]	[0.28–2.06]	[0.27–0.47]	[0.16–0.56]
*Trans*-resveratrol-4′-O-ß-glucuronide	0.64 (100.89)	0.88 (73.75)	0.39 (35.17)	0.05 (210.00)
	[<LLOQ-1.54]	[0.46–2.03]	[0.30–0.60]	[<LLOQ-0.21]
*Trans*-resveratrol-3-O-ß-glucuronide	227.04 (34.98)	111.74 (32.67)	72.43 (41.02)	5.52 (73.52)
	[169.13–342.81]	[64.81–152.02]	[31.32–98.84]	[1.40–10.43]
*Trans*-resveratrol-3-sulfate	36.93 (43.61)	18.64 (27.77)	9.35 (13.50)	1.19 (69.19)
	[24.89–60.12]	[12.43–26.25]	[8.23–11.08]	[0.45–2.12]
*Trans*-resveratrol	5.61 (77.59)	1.25 (75.56)	0.60 (72.28)	0.29 (198.28)
	[2.16–11.51]	[0.41–2.87]	[<LLOQ-1.01]	[<LLOQ-1.15]
DHR-glucuronide-sulfate	/	/	/	8.94 (52.68)
				[4.17–13.64]
DHR-glucuronide	/	/	/	47.31 (30.96)
				[33.68–67.93]
DHR-sulfate	/	/	/	6.98 (20.44)
				[5.77–9.03]

Mean values in µmol.L^−1^. Values expressed as “*trans*-resveratrol-4′-O-ß-glucuronide equivalent” for *trans*-resveratrol glucuronide sulfate and DHR-glucuronide sulfate; as “*trans*-resveratrol-3-O-ß-glucuronide equivalent” for the DHR-glucuronide and as “*trans*-resveratrol-3-sulfate equivalent” for the DHR-sulfate. CV% between mouse lemurs (round brackets), range [square brackets]. There were three determinations for each concentration. *n*: sample size.

LLOQ: lower limit of quantification.

Thus, mouse-lemur plasmas contained monoglucurono-, monosulfo-, di-glucurono-, and both glucurono- and sulfo-conjugated derivatives as the main resveratrol metabolites. All these derivatives have already been detected in mouse [11], pig [14], [30], and human [31], [32] plasmas. Some have been found in the rat [9], [12], [33] and dog [13]. However, results are difficult to compare because of the heterogeneous conditions that have been used for resveratrol administration (see table 3).

**Table 3:** Main resveratrol metabolites after a single dose of pure *trans*-resveratrol administration in different species.

citation	model	administration	Dose	Type of study	Two main metabolites
[11]	mouse	Oral	150 mg.kg^−1^	30 and 60 min after administration	3RG>3RS
[30]	pig	intragastric	6,25 mg.kg^−1^	pharmacokinetic study 0→300 min	3RG>RS
[14]	pig	intragastric	6,25 mg.kg^−1^	6 h after administration	3RG>RS
[43]	Dogs	oral	200 mg.kg^−1^	pharmacokinetic study 0→24 h	RS>RG
		oral	600 mg.kg^−1^	pharmacokinetic study 0→24 h	RG>RS
		oral	1200 mg.kg^−1^	pharmacokinetic study 0→24 h	RG>RS
[33]	rats	oral	50 mg.kg^−1^	pharmacokinetic study 0→24 h	RG≈RS
	rats	oral	150 mg.kg^−1^	pharmacokinetic study 0→24 h	RS>RG
[42]	rats	IV	15 mg.kg^−1^	pharmacokinetic study 0→720 min	RG>RS
[46]	human	Oral	0,5 g→5 g	pharmacokinetic study 0→24 h	3RS>RG

RG = resveratrol-glucuronide; 3RG = *trans*-resveratrol-3-O-glucuronide; RS = *trans*-resveratrol-sulfate; 3RS = *trans*-resveratrol-3-O-sulfate.

In the human gut, part of resveratrol is metabolized into DHR [34]. After resveratrol consumption, this compound [35], [36] and its metabolites [27] can be retrieved from human plasma. They have been also detected in pig [14] and rat [37] tissues. The pseudo-molecular DHR ion (C_14_H_13_O_3_) is the *m/z* 229.087 ion. A low-intensity ion, *m/z* 229.072, was found in data obtained with plasma 1, 2, and 6 h after intake: this corresponded to the m/z+2 of isotopic mass of the 227.072 ion (Fig. 2). Herein, DHR was not present in these mouse lemur plasmas. In contrast, in plasma at 24 h after intake, DHR metabolites were detected.

Figure 4b shows the variation in intensity of the *m/z* 229.086 ion obtained at high-collision energy versus time. This chromatogram shows the presence of one DHR-glucuronide with a retention time of 3.49 min, one DHR-sulfate with a retention time of 3.88 min, and one both glucurono- and sulfo-conjugated derivative with a retention time of ∼2 min. Figure 4b also shows the high-collision energy spectrum at 2.05 min. Ions corresponding to one both glucurono- and sulfo- conjugated derivative are noted *m/z* 485.074 together with *m/z* 309.043 and 405.118, appeared after the loss of the glucurono and sulfo moiety respectively. The daughter ion specific to DHR *m/z* 123.045 was also detected at the same retention time [28]. Table 1 shows the average *m/z* ratio with a satisfactory accuracy value (lower than 10 ppm) for each ion. Identification of these metabolites is not totally complete because our data did not allow us to know the position(s) of the sulfo and/or glucurono moieties.

To sum up, nine resveratrol derivatives were identified in mouse-lemur plasma samples by using UHPLC–Q-TOF–MS^E^ and a semi-targeted approach. High-resolution mass spectrometry, Q-TOF, confirmed the structures of ions and their elemental compositions. These results, obtained by UPLC–Q-TOF–MS^E^, are complementary to those already described in the literature, where compounds have been identified by liquid chromatography coupled to a diode-array detector [12] or a low-resolution mass spectrometer, such as an ion trap [12] or a triple quadrupole [28]. The ion trap confirms the compound presence by displaying the MS^2^ spectrum; the triple quadrupole identifies the molecules according to their specific transitions (parent/daughter ions) in MRM mode [38], [39] and, in our work, Q-TOF confirmed the presence of the compounds by measuring the exact ratio *m/z* of the parent and daughter ions.

### 2. Quantification of resveratrol and its metabolites

A full validation of the quantification method requires a large volume of plasma from untreated lemurs, i.e., ∼15 mL of plasma (30 mL of blood), which would require killing about 10 lemurs: this is a far too high number and poses an ethical problem. Therefore, in this study, we examined the eventual analysis interferences from the grey mouse lemurs plasma by analyzing, with the quantification method [11], the plasma of three lemurs not treated with resveratrol. No interference was present at the retention times for the compounds tested. Thus, we used this quantification method validated in mice and described by Menet at al. [11] to quantify the compounds in the plasma from grey mouse lemurs since the mouse plasma, like that from lemurs, contained no interference that could disturb determination of the compounds

The design of this study was not to carry out a pharmacokinetic study but, rather, to be a preliminary study on resveratrol metabolism in this lemur. Indeed, a complete pharmacokinetic study would have required many more animals as it is impossible to collect more than 150 µL of plasma per animal (i.e., 10% of total plasma volume).

Previous studies in various species have shown that intestinal absorption of free *trans*-resveratrol is complex [40] and is limited by its rapid metabolism in enterocytes to yield glucuronide and sulfate (*trans*-resveratrol-3-O-ß-D-glucuronide and *trans*-resveratrol-3-sulfate) which are subsequently excreted through ABC membrane proteins [41]. In our study, the concentrations of *trans*-resveratrol and its main identified metabolites, *trans*-resveratrol-3-O-ß-D-glucuronide, *trans*-resveratrol-4′-O-ß-D-glucuronide, *trans*-resveratrol-3-sulfate, two *trans*-resveratrol glucuronide sulfates, DHR-glucuronide-sulfate, DHR-glucuronide and DHR-sulfate were determined in the plasma from male mouse-lemurs at 1, 2, 6, and 24 h after intake of 200 mg.kg^−1^ of resveratrol, equivalent to 14 g/70 kg in humans (Table 2).

Free *trans*-resveratrol was present in the plasma in the first hour and 2 h after intake, at concentrations of 5.61 (CV 77.6%) and 1.25 (CV 75.6%) µmol.L^−1^, respectively (Table 2). It also remained measurable after 6 and 24 h. However, *trans*-resveratrol was rapidly metabolized into two main metabolites. The most concentrated metabolite was *trans*-resveratrol-3-O-ß-D-glucuronide, whereas *trans*-resveratrol-3-sulfate was six times less concentrated. The prevalence of these two metabolites has been found in several species, such as mouse [11] and pig [14], [30]. This result is less clear in rat [33], [42] and dog [13], [43] (see Table 3). However, when high doses of the pure resveratrol *trans*-isoform are administered, resveratrol sulfate derivatives have been found to be the main metabolites in humans [32], [38], [39], [44], [45], [46], [47]. Sometimes, glucuronide derivatives have been the major metabolites, but only after low doses of resveratrol intake [38], [48], [49]. However, optimization of bioavailability may favor the presence of the sulfate form at the expense of glucuronide derivatives, even with low doses of resveratrol [50].

The *trans*-resveratrol-4′-O-ß-glucuronide was also present and quantifiable in most mouse-lemur plasmas. The presence of this metabolite has been previously reported in humans. When resveratrol was ingested through moderate consumption of red wine, glucurono conjugation in the 3 or 4′ position has been shown to be significant in metabolism [47], but seems to differ significantly according to the individual [51]. However, *trans*-resveratrol-4′-O-ß-glucuronide in the mouse lemur represents a minor metabolism pathway compared to conjugation in position 3. As already reported in mice [11], the hydroxyl moiety at position 3 is probably the main position for conjugation. Moreover, regarding these data, *trans*-resveratrol glucuronide sulfate derivatives, found in small quantities in plasma, could arise from the sulfo-conjugation of *trans*-resveratrol 3-O-glucuronide and glucurono-conjugation of *trans*-resveratrol-sulfate. Indeed concentrations of these metabolites are highest at 2 h after resveratrol intake, whereas concentrations of 3-mono-conjugated metabolites are highest at 1 h after intake.

DHR was not present whatever the time of collection, and the hydrophilic metabolites of DHR were only found at 24 h after intake at a concentration of 47.3 µmol.L^−1^ (CV 31.0%) for the most concentrated compound, i.e., DHR-glucuronide. DHR is supposed to be a result of metabolism by gut microbiota [34]. Our study did not investigate the metabolites produced between 6 and 24 h after resveratrol absorption. Therefore we cannot exclude the possibility of the presence of DHR in plasma during this period. Of note, an enterohepatic cycle has been evoked for resveratrol in rats [52] and in humans [27], [46], leading to delayed delivery of resveratrol in the gut. Moreover, DHR, when orally administered in rats, is absorbed and metabolized into DHR glucuronide and sulfate [9]. Thus, we can hypothesize that the presence of DHR metabolites in plasma samples only at 24 h was the result of late gut transformation of resveratrol into DHR, of DHR absorption and subsequent DHR metabolism into DHR glucuronide and sulfate. However, resveratrol, particularly when high doses are administered, may, by its antibacterial properties, affect the gut's bacterial profile and thus the rate of gut metabolism [53]. Further studies are needed to investigate these issues.

Intake of resveratrol at 200 mg.kg^−1^ may appear high for a study on metabolism, but this dose has been safety used for a long period in the Restrikal project [20], [21]. Moreover, higher doses have been reported to be well tolerated in rats and rabbits [54], [55]. The dose of 200 mg.kg^−1^ corresponds to about 14 g/70 kg of human bodyweight. Several clinical trials have been performed with good tolerance of doses of up to 5 g per day, which is not far from the 14 g/70 kg of bodyweight given to humans [44], [56]. With high repeated doses, mild to moderate gastrointestinal disorders have been reported in humans, mainly diarrhea [16]. Thus, we cannot exclude that the use of 200 mg.kg^−1^ resveratrol may alter, at least in part, gastrointestinal absorption, which may then impact its metabolism. However, in the design of our study, because each lemur only received one dose, it seems unlikely that this would lead to significant modification of resveratrol metabolism and absorption.

## Conclusion

Resveratrol and its metabolites were identified in *Microcebus murinus* plasma after oral intake of 200 mg.kg^−1^ of resveratrol using UHPLC coupled to a Q-TOF mass spectrometer (Synapt^R^) in MS^E^ mode. Resveratrol treatment led to the presence of quantifiable free resveratrol in the plasma over a 24-h period in the grey mouse lemur. Six hydrophilic metabolites of resveratrol and three hydrophilic metabolites of DHR were identified, and, among them, the two main metabolites were trans-resveratrol-3-O-ß-glucuronide and trans-resveratrol-3-sulfate.

However, although the grey mouse lemur is phylogenetically closer to humans than to rodents and, in many respects, is a relevant model for studying ageing, it appears that its resveratrol-metabolism profile is nearer to mouse than to humans. These data will be useful when interpreting the effect of resveratrol in the mouse lemur and will provide further information about the interspecies characteristics of resveratrol metabolism.
